# Electron density derived from dual-energy CT for predicting thrombolytic therapeutic efficacy in patients with pulmonary embolism

**DOI:** 10.1007/s11604-025-01747-z

**Published:** 2025-02-14

**Authors:** Hiroaki Nagano, Koji Takumi, Erina Nagano, Ryota Nakanosono, Masatoyo Nakajo, Kiyohisa Kamimura, Masanori Nakajo, Fumiko Kanzaki, Fumitaka Ejima, Takuro Ayukawa, Tomohito Hasegawa, Tsubasa Nakano, Mitsuho Hirahara, Takashi Yoshiura

**Affiliations:** 1 Departments of Radiology, Kagoshima University Graduate School of Medical and Dental Sciences, 8-35-1 Sakuragaoka, Kagoshima, 890-8544 Japan; 2 Department of Advanced Radiological Imaging, Kagoshima University Graduate School of Medical and Dental Sciences, 8-35-1 Sakuragaoka, Kagoshima, 890-8544 Japan

**Keywords:** Pulmonary embolism, Dual-energy computed tomography, Thrombolytic therapeutic efficacy, Electron density

## Abstract

**Purpose:**

To clarify the usefulness of electron density (ED) using dual-energy CT (DECT) parameters for predicting treatment response in patients with pulmonary embolism (PE).

**Materials and methods:**

The study population comprised 30 patients with PE (49 thrombi) who underwent pretreatment DECT. The study coordinator diagnosed PE using contrast-enhanced CT (CECT) as the gold standard and annotated the location of thrombi on CECT prior to the DECT image analyses. CT attenuation values on conventional 120 kVp, 40 keV, and 70 keV virtual monochromatic (VM) images; effective atomic number; and ED of pretreatment pulmonary thrombi were measured on unenhanced CT. Thrombi were classified into dissolved and residual groups according to the findings of posttreatment follow-up CT. DECT parameters were compared between the two groups using the Mann–Whitney *U* test. For statistically significant parameters, receiver-operating characteristic (ROC) analysis was used to evaluate their performance for differentiating two groups. Diagnostic accuracy for predicting treatment response in patients with PE was determined by calculating the area under the ROC curve (AUC).

**Results:**

ED values, CT values on conventional 120 kVp imaging, and those on 70 keV VM imaging were significantly higher in thrombi in the dissolved group than the residual group (*p* < 0.001, *p* = 0.012, *p* = 0.009, respectively). AUC values for predicting dissolution response by ED, conventional 120 kVp imaging, and 70 keV VM imaging (cut-off value, 3.49 × 10^23^/cm^3^, 53.4 HU, and 50.7 HU, respectively) were 0.856, 0.744, and 0.755, respectively. AUC was significantly higher for ED than for conventional 120 kVp imaging and 70 keV VM imaging (*p* = 0.032, *p* = 0.016).

**Conclusions:**

ED derived from unenhanced DECT may help predict therapeutic efficacy in patients with PE.

## Introduction

Pulmonary embolism (PE) is a common cardiovascular disorder with a high mortality rate [1]. It has been reported that acute PE develops into chronic thromboembolic pulmonary hypertension in 0.5% to 9.1% of cases [2]. Anticoagulation therapy remains the primary treatment approach for PE; however, some thrombi are resistant to fibrinolytic agents. Computed tomography (CT), especially CT pulmonary angiography (CTPA), is the primary diagnostic tool in the diagnostic algorithm for PE [3]. Measurement of CT attenuation (in HU) of the thrombus has been reported as a useful predictor of the efficacy of anticoagulation therapy for acute ischemic stroke [4, 5]. At present, no study has reported the usefulness of imaging findings for predicting the therapeutic effect of anticoagulant therapy for PE.

Dual-energy CT (DECT) has been introduced into clinical practice and applied in the diagnosis of PE [6]. The iodine map derived from DECT enables quick assessment of perfusion defects in the lung parenchyma caused by PA occlusion [7, 8]. Virtual monochromatic (VM) images can improve the visualization and diagnostic accuracy of PE by increasing vascular attenuation and the contrast-to-noise ratio [9]. Advances in DECT technology now enable the measurement of electron density (ED), a parameter indicating the probability of electron presence in a particular location that is influenced by the tissue’s molecular structure [10, 11]. In clinical practice, previous reports have demonstrated that ED reflects tissue characteristics and is effective in diagnosing glioma, lymph node metastasis in non-small cell lung cancer, head and neck cancer, and gastric cancer [12–15]. ED has the potential to provide additional information regarding changes in a tissue’s elemental composition and may be applicable for evaluating the internal characteristics of blood clots. Therefore, we hypothesized that DECT-derived ED can help predict treatment response in patients with PE. The purpose of this study was to evaluate the usefulness of ED measured by DECT for predicting treatment response in patients with PE.

## Materials and methods

This study was approved and the requirement for informed consent from the study subjects was waived by the institutional review board due to the retrospective study design. This study was conducted in accordance with the Declaration of Helsinki and Ethical Guidelines for Medical and Health Research Involving Human Subjects in Japan.

### Patients

**Fig. 1:**
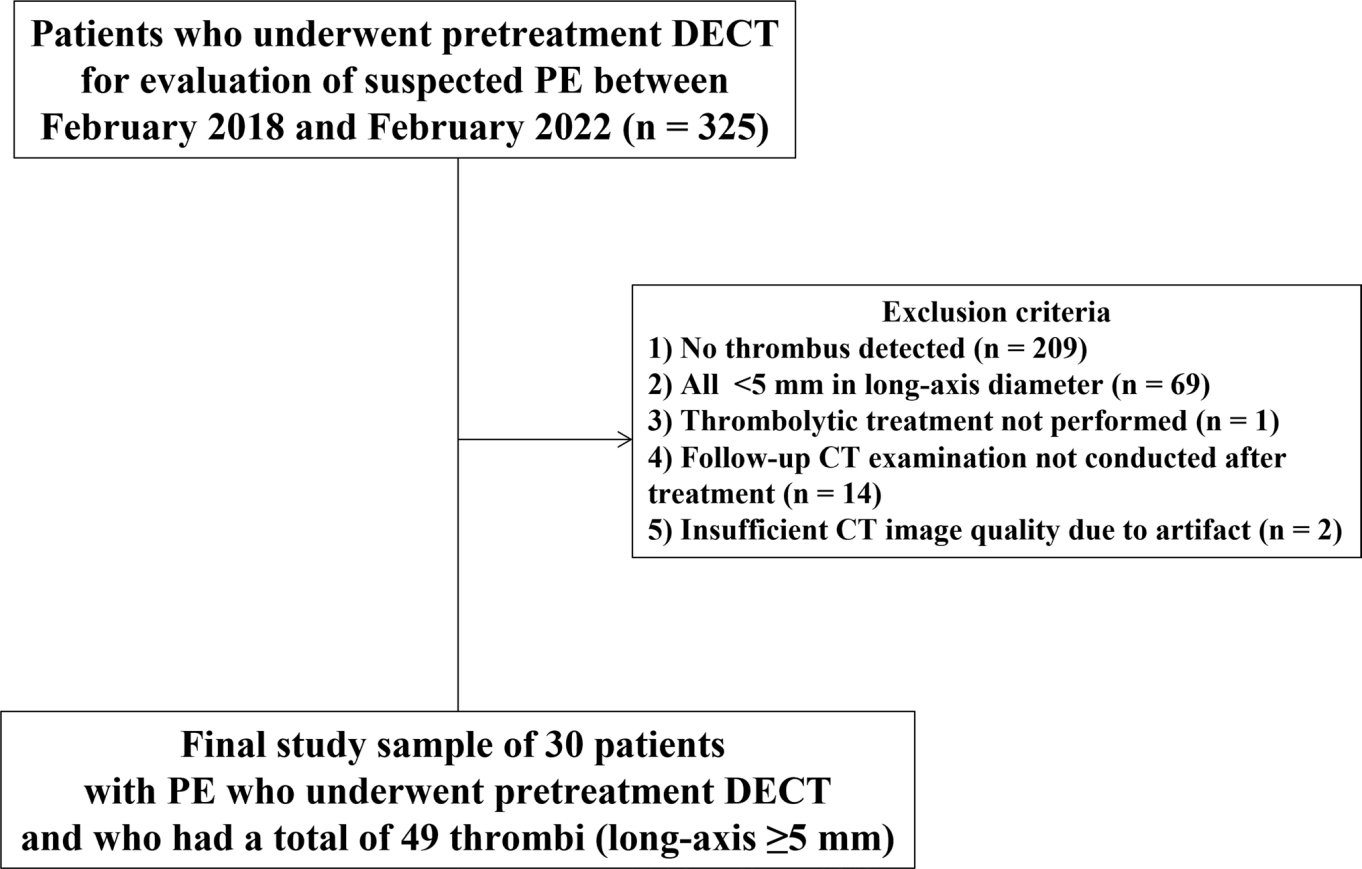
Diagram of the patient selection process. *DECT* dual-energy CT, *PE* pulmonary embolism

A review of the radiology department’s CT database identified 325 patients who had undergone pretreatment CT using DECT between February 2018 and February 2022 for evaluation of suspected PE. Patients were excluded for the following reasons by a study coordinator: (1) no thrombus detected (*n* = 209), (2) all thrombi < 5 mm in long-axis diameter (*n* = 69), (3) thrombolytic treatment not performed (*n* = 1), (4) follow-up CT examination not conducted after treatment (*n* = 14), (5) insufficient CT image quality due to artifact (*n* = 2). Applying these exclusions resulted in a study sample of 30 patients. These 30 patients had a total of 129 thrombi. No more than 2 thrombi per patient were selected, and larger thrombi were selected priority. Among the 129 thrombi, 5 patients had 1 thrombus, 5 patients had 2 thrombi, 4 patients had 3 thrombi, 5 patients had 4 thrombi, 1 patient had 5 thrombi, 3 patients had 6 thrombi, 2 patients had 7 thrombi, 2 patients had 8 thrombi, 1 patient had 9 thrombi, and 2 patients had 10 thrombi. Of the 129 thrombi, 14 (10.9%) were in a main pulmonary artery, 34 (26.4%) were at the lobar level, 56 (43.4%) were at the segmental level, and 25 (19.4%) were at the subsegmental level. A final total of 30 patients with 49 thrombi were included in the study (Fig. 1). We collected patient demographics and clinical characteristics by further review of all patients’ electronic clinical records. All patients were treated with anticoagulants.

### Imaging technique

All CT examinations were performed using a 64-multidetector-row dual-layer DECT system (IQon Spectral CT, Philips Healthcare, Best, The Netherlands) and the scanning protocol included both unenhanced and enhanced scans. We included unenhanced CT in the protocol because previous reports [16] have indicated that unenhanced imaging as part of the CT pulmonary angiography protocol is useful. In emergency settings where PE is suspected, unenhanced CT provides valuable information for the differential diagnosis of other thoracic conditions, such as pulmonary parenchymal or pleural lesions, as well as calcified lesions, including hilar lymph nodes or calcified thrombi. All patients were scanned in the supine position with hands raised above the head. Imaging parameters were as follows: tube voltage, 120 kVp; gantry rotation time, 0.4 s; effective tube current–time product, 160 mAs with auto-modulation; pitch, 0.703; and detector-row configuration, 64 × 0.625 mm. Axial conventional 120 kVp images, 40 and 70 keV VM images, iodine concentration (IC) maps, effective atomic number maps, and ED maps were reconstructed with a slice thickness of 1 mm using an unenhanced CT data set. All dual-energy-based parameters were derived from the two-base model including Compton-scatter-like and photoelectric-like images [17], which in turn were extracted from the low and high-energy data. ED was estimated based on a linear combination of Compton-scatter-like and photoelectric-like images and determined by finding the best fit to the expected ED of tissues corresponding to the energy-dependent attenuation curve created with the aid of the National Institute of Standards and Technology elemental attenuation values [18, 19]. The 40 and 70 keV VM images were reconstructed, and IC, effective atomic number, and ED values were automatically calculated using a thin-client workstation (Spectral Diagnostic Suite; Philips Healthcare). Clinical CTPA scans were obtained with the following contrast agent injection and scan protocol: a nonionic contrast agent (Omnipaque 300 mgI/mL, Daiichi Sankyo, Tokyo, Japan) was injected with a power injector, and scanning was initiated 6 s after attenuation in the pulmonary artery increased to a default threshold (100 HU), as measured by a dedicated monitoring system.

In addition, to evaluate the treatment response of thrombi, all patients underwent follow-up chest contrast-enhanced CT (CECT) examination (median, 94 days; range, 11–734 days) after initiation of anticoagulation therapy.

### Image analysis

A study coordinator (KT, with 23 years of experience in cardiothoracic radiology) diagnosed PE using CECT as the gold standard and annotated the location of the 49 thrombi on CECT prior to the DECT image analyses. DECT image analysis was performed with a thin-client workstation (Spectral Diagnostic Suite, Philips Healthcare). All images were independently evaluated in a random order by two radiologists (EN and HN, with 5 and 12 years of experience in cardiothoracic radiology, respectively) who were blinded to the patients’ clinical course and laboratory findings. The location, level (main, lobar, segmental, or subsegmental pulmonary artery), and extent (occlusive vs non-occlusive) of PE were recorded. PEs were classified as occlusive if no contrast material was visualized distal to the clot. Any discrepancies between the radiologists’ assessments were resolved by consensus. Each radiologist also independently drew a freehand region of interest (ROI) to include as much of the thrombus as possible, on the slice where the thrombus exhibited the largest area on the unenhanced CT images with reference to the CECT images. Mean ROI size was 35.77 ± 43.44 mm^2^ and 41.07 ± 53.15 mm^2^ for radiologists EN and HN, respectively. Care was taken not to include calcification or artifacts within the ROI. If the margin of the thrombus was unclear, then the ROI was drawn slightly inside the apparent margin of the thrombus. The copy-and-paste function was used to transfer the ROI from the conventional 120 kVp images to the 40 and 70 keV VM images and to each of the IC, effective atomic number, and ED maps. The mean ROI area was recorded for each DECT parameter.

### Evaluation of anticoagulation response

ROIs were also placed on posttreatment CT images for the measurement and evaluation of thrombi. Treatment response for thrombus was assessed by comparing ROI size between the pretreatment and posttreatment follow-up CECT images, using the following 5-point thrombolytic therapeutic efficacy scoring system: 0, increase; 1, decrease 0–20%; 2, decrease 20–50%; 3, decrease over 50%; and 4, complete resolution. Patients were divided into two groups according to response score: dissolved group, scores 3–4; and residual group, scores 0–2.

### Statistical analysis

Data are presented as percentages or as the mean and SD. Interobserver agreement of quantitative analyses was evaluated using the intraclass correlation coefficient (ICC), (0.00–0.20, poor correlation; 0.21–0.40, fair correlation; 0.41–0.60, moderate correlation; 0.61–0.80, good correlation; 0.81–1.00, excellent correlation) and Bland–Altman plot. Mean attenuation (for conventional 120 kVp and 40 and 70 keV VM images), IC, effective atomic number, and ED were compared between the dissolved and residual groups using Mann–Whitney *U* test.

Receiver-operating characteristic (ROC) analysis was performed among variables that showed significant differences between the dissolved and residual groups. The optimal threshold criterion for predicting therapeutic efficacy was determined by the Youden index. Sensitivity, specificity, positive predictive value, negative predictive value, and accuracy for predicting thrombolytic therapeutic efficacy were calculated for each parameter. Diagnostic accuracy was determined by calculating the area under the ROC curve (AUC). DeLong method was used for comparison of AUCs. P < 0.05 indicated a statistically significant difference in all analyses. Statistical analyses were performed using SPSS 29.0 software and MedCalc 22.003 software.

## Results

### Patients and thrombi

A total of 30 consecutive patients (10 men, 20 women; mean age, 61.4 ± 16.1 years; age range, 30–88 years) were enrolled in this study (Table 1**)**. All patients received anticoagulant therapy using either vitamin K (*n* = 17) or a direct oral anticoagulant (n = 13).

**Table 1:** Patient characteristics (*n* = 30)

Characteristic	Value
Age (y), mean ± SD (range)	61.4 ± 16.1 (30–88)
Sex	
Male	10 (33)
Female	20 (67)
History	
Smoking	14 (47)
Hypertension	10 (33)
Diabetes mellitus	3 (10)
Malignancy	7 (23)
Deep venous thrombosis	17 (57)
Recent surgery	3 (10)
Prolonged immobilization	4 (13)

Except where indicated, data are presented as the number of patients (percentage)

Among the 49 thrombi, 11 (22.4%) were in a main pulmonary artery, 13 (26.5%) were at the lobar level, 23 (46.9%) were at the segmental level, and 2 (4.1%) were at the subsegmental level. Seven (14.3%) thrombi were occlusive and 42 (85.7%) were non-occlusive. Table 2 shows the distribution of PEs by location, level, and extent.

**Table 2:** Characteristics of pulmonary embolisms detected by CT angiography (*n* = 49)

Characteristic	Value
Location of pulmonary embolism	
Right main	9 (18)
Left main	2 (4)
Right upper lobe	6 (12)
Right middle lobe	2 (4)
Right lower lobe	15 (31)
Left upper lobe	3 (6)
Left lower lobe	12 (24)
Level of pulmonary embolism	
Main	11 (22)
Lobar	13 (27)
Segmental	23 (47)
Subsegmental	2 (4)
Extent of pulmonary embolism	
Occlusive	7 (14)
Non-occlusive	42 (86)

Except where indicated, data are presented as the number of patients (percentage)

### Interobserver agreement

Interobserver agreement ranged from moderate (ICC = 0.72) to excellent (ICC = 0.94) for quantitative measurements. ED exhibited excellent interobserver agreement (ICC = 0.94).

Bland–Altman analyses showed a small bias and narrow 95% limits of agreement for the quantitative parameters (Table 3). For ED, the bias was –0.06, and the 95% limits of agreement were – 0.9 and + 0.8.

**Table 3:** Interobserver agreement

Quantitative parameter	ICC	Bland–Altman analysis (%)		
			95% limits of agreement	
		Bias	Lower	Upper
Attenuation (120 kVp images)	0.88 (0.80, 0.93)	– 1.4	– 14.6	11.7
Attenuation (40 keV VM images)	0.83 (0.72, 0.90)	– 2.3	– 20.3	15.6
Attenuation (70 keV VM images)	0.91 (0.85, 0.95)	– 0.8	– 11.5	9.9
Iodine concentration	0.72 (0.55, 0.83)	– 0.02	– 0.25	0.21
Effective atomic number	0.78 (0.63, 0.87)	– 0.01	– 0.15	0.13
Electron density	0.94 (0.89, 0.96)	– 0.06	– 0.93	0.81

Values are expressed as the kappa coefficient for qualitative parameters and as the intraclass correlation coefficient for quantitative parameters (95% Cis)

*ICC* intraclass correlation coefficient, *VM* virtual monochromatic

### Comparison of DECT parameters between the dissolved and residual groups

Compared with the residual group, the dissolved group exhibited significantly higher attenuation on 120 kVp images (55.75 ± 13.75 vs 44.47 ± 7.41 HU, *p* = 0.012), higher attenuation on 70 keV VM images (56.13 ± 13.22 vs 45.16 ± 7.94 HU, *p* = 0.009), and higher ED (3.52 ± 0.04 × 10^23^/cm^3^ vs 3.48 ± 0.02 × 10^23^/cm^3^, *p* < 0.001) (Table 4). There was no significant difference between the groups in terms of attenuation on 40 keV VM images, IC, or effective atomic number (all *p* > 0.05).

**Table 4:** Comparison of DECT parameters between the dissolved and residual groups

Characteristic	Dissolved group (*n* = 37)	Residual group (*n* = 12)	p
Attenuation (HU, 120 kVp images)	55.75 ± 13.75	44.47 ± 7.41	.012
Attenuation (40 keV VM images)	59.22 ± 15.91	50.93 ± 13.29	.156
Attenuation (70 keV VM images)	56.13 ± 13.22	45.16 ± 7.94	.009
Iodine concentration	0.10 ± 0.13	0.16 ± 0.14	.278
Effective atomic number	7.30 ± 0.09	7.33 ± 0.11	.235
Electron density (× 10^23^/cm^3^)	3.52 ± 0.04	3.48 ± 0.02	< .001

Data are presented as the mean ± SD

*VM* virtual monochromatic

### AUC for diagnostic accuracy of ED, 70 keV VM, and CT attenuation (120 kVp) in differentiation of dissolved and residual thrombi

**Fig. 2:**
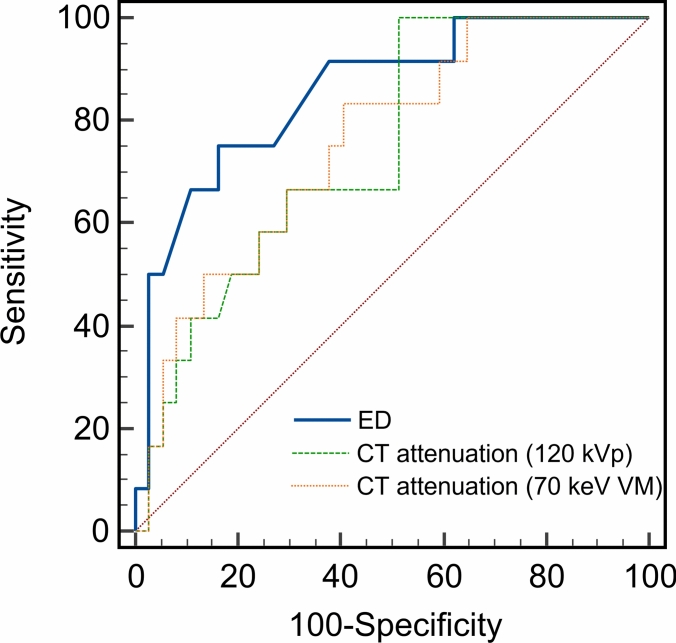
Receiver-operating characteristic curves for electron density (ED) and CT attenuation (120 kVp and 70 keV virtual monochromatic images) for differentiation of dissolved and residual thrombi

**Fig. 3:**
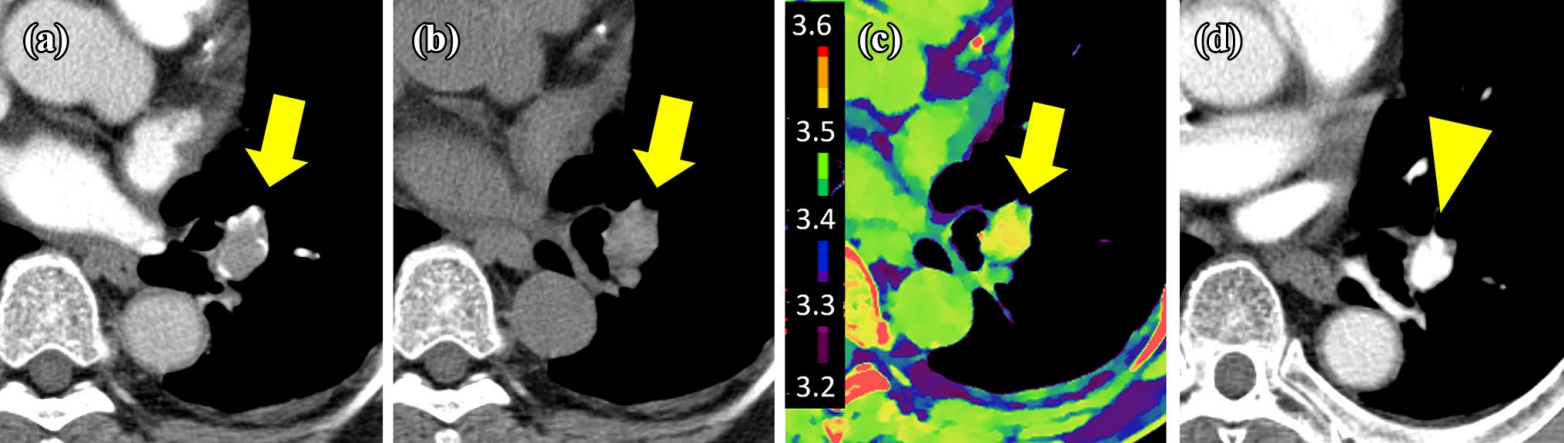
Representative images in the dissolved group. A 72-year-old male with thrombus (arrows) located in the left lower lobe pulmonary artery. The thrombus is seen on pretreatment CECT (**a**), and demonstrates slightly high attenuation on conventional unenhanced CT (120 kVp) (**b**) and a high ED value (3.54 × 10^23^/cm^3^) (**c**). Follow-up CECT (**d**) shows complete resolution of the thrombus (arrowhead) after treatment (therapeutic efficacy score: 4) and recovery of blood flow

**Fig. 4:**
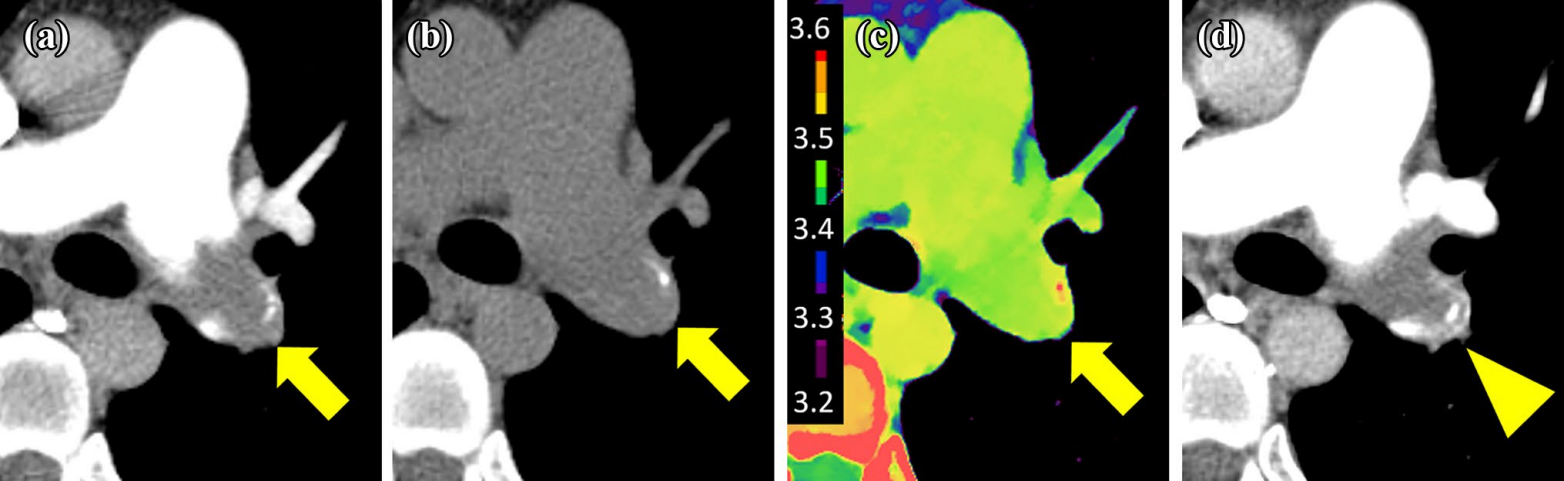
Representative images in the residual group. A 45-year-old male with thrombus (arrows) in the left main pulmonary artery trunk. The thrombus is seen on pretreatment CECT (**a**), but cannot be detected on unenhanced CT (120 kVp) (**b**). It has an ED value of 3.49 × 10^23^/cm^3^ (**c**). Follow-up CECT (**d**) shows residual thrombus (arrowhead) after treatment (therapeutic efficacy score: 1, decrease of 16.8%)

Optimal threshold values for differentiating dissolved and residual thrombi were identified as ≤ 53.4 HU for CT attenuation on 120 kVp images, ≤ 50.7 HU for CT attenuation on 70 keV VM images, and ≤ 3.49 × 10^23^/cm^3^ for ED. The sensitivity, specificity, and AUC for CT attenuation on 120 kVp images were 48.6%, 100%, and 0.744, respectively; those for CT attenuation on 70 keV VM images were 59.5%, 83.3%, and 0.755, respectively; and those for ED were 83.8%, 75.0%, and 0.86, respectively (Fig. 2). AUC was significantly higher for ED than for CT attenuation (120 kVp or 70 keV VM images) (p = 0.032 and = 0.016, respectively). Representative cases of dissolved and residual thrombi are shown in Figs. 3, 4.

## Discussion

The major finding of the present study is that ED obtained from DECT is helpful for predicting treatment response in patients with PE. Thrombi with higher ED values were associated with better treatment outcomes. To the best of our knowledge, the potential role of ED in predicting therapeutic efficacy in patients with PE has not been previously demonstrated. ED may have potential as a new biomarker for predicting response to PE treatment.

PE commonly originate from deep vein thrombi, which are composed mainly of erythrocytes with large amounts of fibrin and relatively few platelets [20–22]. Erythrocyte-rich thrombi show higher CT attenuation than platelet-rich thrombi because CT attenuation has a linear correlation with the concentration of hemoglobin [5, 23]. Previous studies have shown that thrombi with higher CT attenuation and rich erythrocyte content are more susceptible to fibrinolytic agents [4, 5]. Thus, it has been reported that CT attenuation of thrombi can predict the outcome and success of anticoagulation therapy [24–27]. However, the association between CT attenuation of thrombi and treatment outcome demonstrates high specificity but is limited by low sensitivity, particularly due to the varying composition of the thrombus [28, 29]. Similarly, the present result showed high specificity (100%) and low sensitivity (48%) for a cutoff value of 53.4 HU.

A previous study has reported the utility of non-contrast DECT for thrombus characterization, with high accuracy for differentiating between RBC-rich and fibrin-rich in-vitro thrombi [30]. In our results, a subtle yet non-significant inclination was observed for the 70 keV VM images from DECT (equivalent to conventional 120 kVp images) to manifest a superior AUC for predicting therapeutic effectiveness in comparison to conventional CT. This finding could be attributed to the ability of VM images derived from DECT to reduce beam hardening artifacts and noise compared to conventional CT images. As such, analysis of processed images derived from DECT (such as VM images) might provide more accurate measurements of the CT values of thrombi, potentially contributing to improved diagnostic capabilities.

In conventional CT, the CT attenuation value indicates the degree of X-ray absorption during passage through an object. Using DECT, however, it becomes feasible to assess the proportion between two phenomena within the X-ray absorption mechanism, specifically photoelectric absorption and Compton scattering, which depend on the X-ray energy as well as the effective atomic number and ED of the tissue. By measuring X-ray attenuation at two different energies, DECT enables the estimation of the effective atomic number and ED of each voxel. Bae et al. [31] have recently shown that compared to conventional CT, ED images can improve the visualization of thrombus in patients with acute pulmonary embolism. In addition, ED has been reported to enable the identification of arterial and intracardiac thrombi that were undetectable using conventional non-contrast CT imaging [32, 33]. In other words, ED has the potential to provide information about the characteristics of thrombi that cannot be evaluated using conventional CT imaging. It has been reported that ED maps can be used to describe different types of atoms, chemical bonds, and tissue compositions, which suggests that they may serve as indicators of alterations in the elemental composition of tissues [13, 34]. Sedaghat et al. [35] have shown that the heightened hemoglobin content at the site of a hematoma increases ED at this location, with a minimal effect on the average atomic number. These findings suggest that ED images could improve hematoma detection. In the present study, the ED values of thrombi derived from unenhanced DECT were significantly higher in the dissolved group than in the residual group. In addition, ED had a significantly higher AUC than CT attenuation (120 kVp). Higher ED in dissolved thrombi may reflect higher erythrocyte content, and may possibly also provide information about the characteristics of thrombi, such as fibrin fiber density, that cannot be evaluated using conventional CT imaging.

The present study had several potential limitations. A primary limitation was the small number of PE patients included from a single institute. In addition, we did not account for possible clustering effects among the two thrombi evaluated in individual patients. Nevertheless, this is the first study to focus on the feasibility of ED measurement to predict therapeutic efficacy in patients with PE. We believe that this pilot study encourages future research including a large multicenter prospective study.

In conclusion, ED values were significantly higher for thrombi in the dissolved group than in the residual group. The application of ED, derived from dual-energy analysis of unenhanced CT, may aid in predicting therapeutic efficacy in patients with PE.

## Abbreviations

PE: Pulmonary embolism
CT: Computed tomography
CTPA: CT pulmonary angiography
DECT: Dual-energy CT
VM: Virtual monochromatic
ED: Electron density
IC: Iodine concentration
CECT: Contrast-enhanced CT
ROI: Region of interest
ROC: Receiver-operating characteristic
AUC: Area under the ROC curve
ICC: Intraclass correlation coefficient
